# Supplementary material for: Clinical and Molecular Spectrum of Sporadic Vascular Malformations: A Single-Center Study
Source: Biomedicines. 2022 Jun 20;10(6):1460. doi: 10.3390/biomedicines10061460 (PMC9220263; doi:10.3390/biomedicines10061460)
Supplement: Supplementary file 1 [file biomedicines-10-01460-s001.zip › biomedicines-1597195-supplementary.pdf]

Table S1. List of genes included in the custom panel for NGS targeted resequencing of vascular anomalies.

ABCC6	*ANTXR1*	*DUSP5*	*FGFR3*	*GNA11*	*IDH2*	*MAPK1*	*PIEZO1*	*RAC1*	*SMO*
ACTB	*ARAF*	*EGFR*	*FLT3*	*GNA14*	*JAK2*	*MET*	*PIK3CA*	*RAF1*	*SOX18*
ACVR1	*ATP2B2*	*ELMO2*	*FLT4*	*GNAQ*	*KDR*	*MTOR*	*PIK3CD*	*RASA1*	*STAMBP*
ACVRL1	*BRAF*	*ENG*	*FOS*	*GNAS*	*KIF11*	*MYC*	*PIK3R1*	*RET*	*STAT3*
ADAMTS3	*CALCRL*	*EPHB4*	*FOSB*	*H3-3A*	*KIT*	*MYCN*	*PIK3R2*	*RHOA*	*STK11*
AGGF1	*CCBE1*	*ERBB2*	*FOXC2*	*H3-3B*	*KRAS*	*NEK9*	*PLCG1*	*ROS1*	*TEK*
AKT1	*CCM2*	*ERBB4*	*GATA2*	*H3C2*	*KRIT1*	*NOTCH1*	*POLE*	*RRAS*	*TERT*
AKT2	*CTNNB1*	*FAT4*	*GDF2*	*H3C3*	*MAP2K1*	*NRAS*	*PTEN*	*RRAS2*	*TP53*
AKT3	*DDR2*	*FBXW7*	*GJC2*	*HRAS*	*MAP2K3*	*PDCD10*	*PTPN11*	*SEC23B*	*VEGFC*
ALK	*DICER1*	*FGFR2*	*GLMN*	*IDH1*	*MAP3K3*	*PDGFRA*	*PTPN14*	*SMAD4*	

Table S2. Minor allele frequencies in gnomAD of pathogenic and undescribed variants identified in our series.

**Gene (RefSeq)**	**Variant(s)***	**Patient n.**	**Reference**	**MAF** in gnomAD browser**
GNA11 (NM_002067.5)	c.547C>T (p.Arg183Cys)	20, 21	[1]	Variant not found
GNA11 (NM_002067.5)	c.548G>A (p.Arg183His)	22	[2]	Variant not found
GNAQ (NM_002072.5)	c.548G>A (p.Arg183Gln)	1-10	[3]	Variant not found
KRAS (NM_033360.4)	c.35G>T (p.Gly12Val)	11	[4]	Variant not found
KRAS (NM_033360.4)	c.183A>T (p.Gln61His)	43	[4]	Variant not found
PIK3CA (NM_006218.4)	c.1636C>A (p.Gln546Lys)	12	[5]	Variant not found
PIK3CA (NM_006218.4)	c.353G>A (p.Gly118Asp)	13, 25	[5]	Variant not found
PIK3CA (NM_006218.4)	c.1090G>A (p.Gly364Arg)	14	[5]	Variant not found
PIK3CA (NM_006218.4)	c.1133G>A (p.Cys378Tyr)	15, 23	[5]	Variant not found
PIK3CA (NM_006218.4)	c.1093G>A (p.Glu365Lys)	16	[5]	Variant not found
PIK3CA (NM_006218.4)	c.1357G>A (p.Glu453Lys)	17, 18	[5]	Variant not found
PIK3CA (NM_006218.4)	c.2740G>A (p.Gly914Arg)	19	[5]	Variant not found
PIK3CA (NM_006218.4)	c.311C>T (p.Pro104Leu)	24	[5]	Variant not found
PIK3CA (NM_006218.4)	c.2176G>A (p.Glu726Lys)	26, 29	[5]	Variant not found

PIK3CA (NM_006218.4)	c. 3132T>G (p.Asn1044Lys)	27	[5]	Variant not found
PIK3CA (NM_006218.4)	c.241G>A (p.Glu81Lys)	28	[5]	Variant not found
PIK3CA (NM_006218.4)	c.344G>C (p.Arg115Pro)	30	[5]	Variant not found
PIK3CA (NM_006218.4)	c.3140A>G (p.His1047Arg)	31, 33, 38	[5]	0.000004028
PIK3CA (NM_006218.4)	c.3073A>G (p.Thr1025Ala)	32	[6]	Variant not found
PIK3CA (NM_006218.4)	c.3140A>T (p.His1047Leu)	35	[5]	0.000004028
PIK3CA (NM_006218.4)	c.325_327delGAA (p.Glu109del)	34,37	[7]	Variant not found
PIK3CA (NM_006218.4)	c.1633G>A (p.Glu545Lys)	36, 39	[5]	0.000004032
***RASA1* (NM_002890.2)**	**c.1570_1571insTA (p.Cys525fs*19)**	**42**	**This study**	**Variant not found**
TEK (NM_000459.4)	c.2690A>G (p.Tyr897Cys)	40	[8]	Variant not found
***TEK* (NM_000459.4)**	**c.2800T>C (p.Ser934Pro)**	**40**	**This study**	**Variant not found**
TEK (NM_000459.4)	c.2690A>T (p.Tyr897Phe)	41	[9]	Variant not found
***TEK* (NM_000459.4)**	**c.2743C>A (p.Arg915Ser)**	**41**	**This study**	**Variant not found**

* Previously undescribed variants are indicated in bold, ** MAF, minor allele frequency.

References

1. Couto, J.A.; Ayturk, U.M.; Konczyk, D.J.; Goss, J.A.; Huang, A.Y.; Hann, S.; Reeve, J.L.; Liang, M.G.; Bischoff, J.; Warman, M.L.; et al. A somatic GNA11 mutation is associated with extremity capillary malformation and overgrowth. *Angiogenesis* **2017**, *20*, 303–306. https://doi.org/10.1007/s10456-016-9538-1.
2. Jordan, M.; Carmignac, V.; Sorlin, A.; Kuentz, P.; Albuisson, J.; Borradori, L.; Bourrat, E.; Boute, O.; Bukvic, N.; Bursztejn, A.C.; et al. Reverse Phenotyping in Patients with Skin Capillary Malformations and Mosaic GNAQ or GNA11 Mutations Defines a Clinical Spectrum with Genotype-Phenotype Correlation. *J. Investig. Dermatol* **2020**, *140*, 1106–1110 e1102. https://doi.org/10.1016/j.jid.2019.08.455.
3. Martins, L.; Giovani, P.A.; Rebouças, P.D.; Brasil, D.M.; Neto, F.H.; Coletta, R.D.; Machado, R.A.; Puppin-Rontani, R.M.; Jr., F.H.N.; Kantovitz, K.R. Computational analysis for GNAQ mutations: New insights on the molecular etiology of Sturge-Weber syndrome. *J. Mol. Graph. Model.* **2017**, *76*, 429–440, https://doi.org/10.1016/j.jmgm.2017.07.011.
4. Al-Olabi, L.; Polubothu, S.; Dowsett, K.; Andrews, K.A.; Stadnik, P.; Joseph, A.P.; Knox, R.; Pittman, A.; Clark, G.; Baird, W.; et al. Mosaic RAS/MAPK variants cause sporadic vascular malformations which respond to targeted therapy. *J. Clin. Investig.* **2018**, *128*, 1496–1508. https://doi.org/10.1172/JCI124649.
5. Kuentz, P.; St-Onge, J.; Duffourd, Y.; Courcet, J.B.; Carmignac, V.; Jouan, T.; Sorlin, A.; Abasq-Thomas, C.; Albuisson, J.; Amiel, J.; et al. Molecular diagnosis of PIK3CA-related overgrowth spectrum (PROS) in 162 patients and recommendations for genetic testing. *Genet. Med.* **2017**, *19*, 989–997. https://doi.org/10.1038/gim.2016.220.
6. Keppler-Noreuil, K.M.; Rios, J.J.; Parker, V.E.; Semple, R.K.; Lindhurst, M.J.; Sapp, J.C.; Alomari, A.; Ezaki, M.; Dobyns, W.; Biesecker, L.G. PIK3CA -related overgrowth spectrum (PROS): Diagnostic and testing eligibility criteria, differential diagnosis, and evaluation. *Am. J. Med Genet. Part A* **2014**, *167*, 287–295, https://doi.org/10.1002/ajmg.a.36836.
7. Blesinger, H.; Kaulfuß, S.; Aung, T.; Schwoch, S.; Prantl, L.; Rössler, J.; Wilting, J.; Becker, J. PIK3CA mutations are specifically localized to lymphatic endothelial cells of lymphatic malformations. *PLoS ONE* **2018**, *13*, e0200343, https://doi.org/10.1371/journal.pone.0200343.

8. Soblet, J.; Kangas, J.; Natynki, M.; Mendola, A.; Helaers, R.; Uebelhoer, M.; Kaakinen, M.; Cordisco, M.; Dompmartin, A.; Enjolras, O.; et al. Blue Rubber Bleb Nevus (BRBN) Syndrome Is Caused by Somatic TEK (TIE2) Mutations. *J. Investig. Dermatol.* **2017**, *137*, 207–216. https://doi.org/10.1016/j.jid.2016.07.034.
9. Limaye, N.; Wouters, V.; Uebelhoer, M.; Tuominen, M.; Wirkkala, R.; Mulliken, J.B.; Eklund, L.; Boon, L.M.; Vikkula, M. Somatic mutations in angiopoietin receptor gene TEK cause solitary and multiple sporadic venous malformations. *Nat. Genet.* **2008**, *41*, 118–124, https://doi.org/10.1038/ng.272.

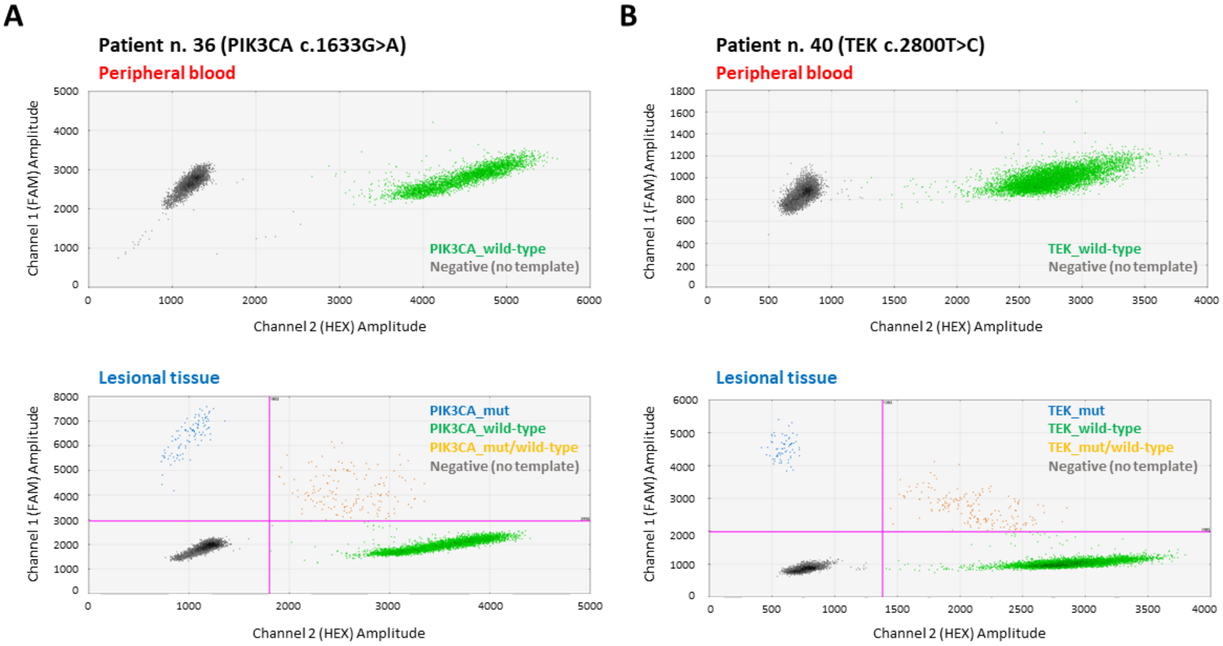

Figure S1. Validation by droplet digital PCR analysis of selected sequence variants in the *PIK3CA* and *TEK* genes.

Droplet digital PCR analysis: (A) Two-dimensional (2D) droplet digital PCR (ddPCR) plots and detection thresholds demonstrating the presence of the known *PIK3CA* mutation c.1633G>A in the genomic DNA obtained from lesional skin of patient n. 36 (VAF: 3.1%), and its absence in the genomic DNA from peripheral blood. Similar findings were obtained for patient n. 39, who carries the same somatic mutation c.1633G>A in the *PIK3CA* gene, in genomic DNA from lesional skin (VAF: 0.83%) (data not shown). (**B)** 2D ddPCR plots and detection thresholds demonstrating the presence of the previously undescribed *TEK* variant c.2800T>C in genomic DNA obtained from lesional skin of patient n. 40 (VAF: 1.5%). Conversely, the mutant allele was undetectable in genomic DNA obtained from the patient's peripheral blood. FAM-labeled oligonucleotides probes target mutant alleles, whilst HEX-labeled oligonucleotide probes target the wild-type alleles. Each ddPCR run included a no-template control, the patient's genomic DNA from peripheral blood as a negative control, and genomic DNA from lesional tissues.

Droplet digital PCR (ddPCR) reactions were performed in a 20 µL reaction mix, containing 1X ddPCR Supermix (no dUPT) (Bio-Rad, Hercules, CA), 900nM primers, 250 nM of each probe, and 100 ng of genomic DNA from lesional tissue or peripheral blood for each sample tested. The wet-lab-validated assay dHsaMDV2010075 (Bio-Rad) was used to detect the known *PIK3CA* mutation c.1633G>A (p.Glu545Lys) of patients n. 36 and 39 (Table 1), whilst a custom assay (Assay ID: dHsaMDS265067620, Bio-Rad) was designed by Bio-Rad proprietary computational algorithms to confirm the presence of the somatic variant c.2800T>C in the *TEK* gene (patient n. 40, Table 1). Droplets were generated and analyzed using the QX200 Droplet Digital PCR System (Bio-Rad). The following thermal cycling conditions were used: (i) 1 cycle at 95°C (2°C/sec ramp) for 10 minutes, (ii) 40 cycles at 94°C (2°C/sec ramp) for 30 seconds and at 55°C (*PIK3CA*) or 52°C for 1 minute (*TEK*), followed by (iii) 1 cycle at 98°C (2°C/sec ramp) for 10 minutes. The sample was held at 4°C until further processing. ddPCR absolute quantifications of mutant and wild-type alleles were estimated by modeling as a Poisson distribution using QuantaSoft Analysis Software v.1.7.4 (Bio-Rad).

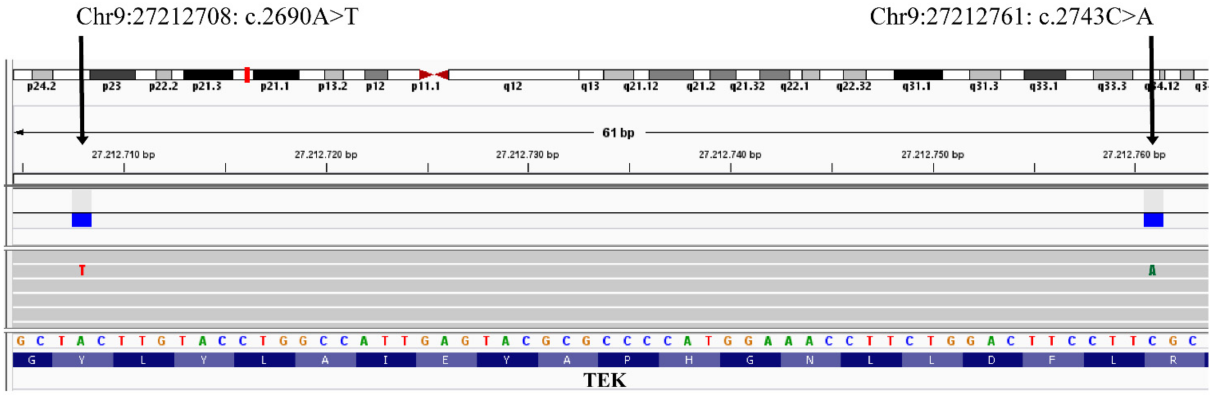

Figure S2. Molecular genetic testing of patient n. 41. Next-generation sequencing (NGS) singleton analysis shows the presence of the variants c.2690A>T (p.Tyr897Phe) and c.2743C>A (p.Arg915Ser) on the same read of the *TEK* gene (NM_000459) in patient n. 41.
